# Indications, Challenges, and Characteristics of Successful Implementation of Perioperative Registries in Low Resource Settings: A Systematic Review

**DOI:** 10.1007/s00268-023-06909-6

**Published:** 2023-01-19

**Authors:** Fitsum Kifle, Tewodros Kifleyohanes, Jolene Moore, Ayele Teshome, Bruce M. Biccard

**Affiliations:** 1 Division of Global Surgery, University of Cape Town (UCT), Cape Town, South Africa; 2 College of Medicine, Asrat Weldyes Health Science Campus, Debre Berhan University, Debre Berhan, Ethiopia; 3 School of Medicine, Medical Sciences and Nutrition, University of Aberdeen, Aberdeen, UK; 4 African Perioperative Research Group, Network for Perioperative and Critical Care (APORG-N4PCc), Addis Ababa, Ethiopia; 5 Ministry of Health, Addis Ababa, Ethiopia; 6 Department of Anaesthesia and Perioperative Medicine, Groote Schuur Hospital, University of Cape Town, Cape Town, Western Cape South Africa

## Abstract

Perioperative registries can be utilised to track outcomes, develop risk prediction models, and make evidence-based decisions and interventions. To better understand and support initiatives to establish clinical registries, this study aimed to assess the indications, challenges, and characteristics of successful perioperative registries in low-resource settings, where there is unmet surgical demand and patients have a mortality rate up to double that of high-income countries. We conducted a librarian-assisted literature search of international research databases of articles published between January 1969 and January 2021. Studies were filtered using predefined criteria and responses to two Mixed Method Appraisal Tool screening questions. A Direct Content Analysis Method was used to synthesis. e data for eligible studies based on predefined criteria. The search identified 2793 abstracts. After removing duplicates and excluding studies that did not meet eligibility criteria, twelve studies were included, conducted in South America (*n* = 4), Africa (*n* = 5), the Middle East (*n* = 2), and Asia (*n* = 1). The lack of context-specific data for determining and evaluating patient outcomes (*n* = 7) was the major indication for implementation. Organising local research teams and engaging stakeholders in the host country were associated with successful implementation. Inadequate funding for data collectors and monitoring data quality were identified as challenges (*n* = 4). The goal of a perioperative registry is to generate data to influence and support quality improvement, and national surgical policies. Efforts to establish perioperative registries in low- and middle-income countries should engage local teams and stakeholders and seek to overcome challenges in data collection and monitoring.

**Supplementary Information:**

The online version contains supplementary material available at 10.1007/s00268-023-06909-6.

## Introduction

Digitalisation, the rise of artificial intelligence, big data analytics, cloud storage, and machine learning have all changed the structure of the information sector, making data the most valuable resource available today [1]. In healthcare, effective data storage can be used to reduce healthcare costs, improve the quality of care, forecast epidemic outbreaks, and help to avoid preventable diseases [2, 3]. The use of clinical registries and data-driven decision-making and policy implementation has become ubiquitous in developed countries, assisting in improving the quality of healthcare and research initiatives [4]. Clinical registries are defined as datasets designed with insight from the surgical provider community to improve and/or inform care. However, limited access to perioperative registries compromises these improvements in low-resource healthcare countries, aggravating global health and data disparities.

Global surgery accounts for 30% of the global health burden [5]. Low-income countries (LICs) are underserviced to provide surgical services, contributing only 3.5% of the global surgical volume, but with significantly higher mortality and morbidity [6, 7]. To improve the access, safety, and overall quality of surgical and anaesthetic care, continuous local data are required for feedback and auditing. Most of the current data from low- and middle-income countries (LMICs) are dependent on either short-term data collection, e.g. the African Surgical Outcome Study (ASOS) [8, 9], or data from predominantly high-income countries (HICs) without much involvement from the data owner LMICs, further exacerbating global inequalities in patient care and research capacity.

This study aimed to determine the indications, challenges, and characteristics necessary for establishing and implementing locally owned perioperative registries in LMICs by systematically reviewing the literature. The objective of the review was to determine the evidence needed for researchers and clinicians to implement perioperative registries in low-resource settings to strengthen local research capacity and improve patient care.

## Materials and methods

The International Prospective Register of Systematic Reviews (PROSPERO) database was checked to ensure that a similar study had not been previously conducted, and the protocol was registered (CRD42021265077) and is available at https://www.crd.york.ac.uk/prospero/display_record.php?ID=CRD42021265077. The PICO (population, intervention, comparator, and outcome) model was used to frame the research questions; *P* = Low- and middle-income nations, population: low- and middle-income countries, Intervention: implementation of perioperative registry networks, Comparison: none, Outcomes: indications, challenges, and characteristics of successful registries.

A librarian assisted with developing an inclusive literature search in accordance with the Preferred Reporting Items for Systematic Reviews and Meta-Analyses (PRISMA) [10], and Assessing the Methodological Quality of Systematic Reviews (AMSTAR) [11] guidelines of five international databases: PubMed, Scopus, Cochrane Library, Web of Science, and WHOLIS WHO Library Database. The review covered articles published between and including January 1969 and January 2021.

The search was conducted using controlled medical subject headings (MeSHs) and keywords for each database: “(Registry or registries),” “Perioperative medicine”, “Perioperative medicine”, or “perioperative care”, or “perioperative care” or “surgery” or “surgical”, and LIC/LMICs filters as per World Bank classification [12]. The search strategy can be found in supplemental file 1. The inclusion criteria were quantitative, qualitative, or mixed-method studies of the implementation of a perioperative registry network or/and hospitals with a perioperative/surgical registry which presented the study outcomes of indications, needs, and challenges in LMICs. We included studies published in all languages. The reference lists of included studies were screened for other eligible studies.

We excluded studies where the primary registry implementation research was not published in peer-review journals (i.e. conference papers, commentaries, letters to the editor, editorials, opinion, discussion, case reports, review studies, meta-analyses, and other secondary studies), studies for which the full text was not available, and studies for which the either or both screening questions, (i.e. “Do the collected data allow us to address the research questions?” and “Are there clear research questions?”) were negative on the Mixed Methods Appraisal Tool (MMAT) [13]. The Mixed Methods Appraisal Tool is a checklist for simultaneously evaluating and/or describing research in systematic mixed studies (reviews including original qualitative, quantitative, and mixed methods studies). The abstracts were screened by FK, TK, and JM using the MMAT, with any inconsistencies resolved through discussion with BB.

The following variables were extracted to a spreadsheet from included publications: authors, publication year, registry objective(s), country of study, study design, the language of study, and MMAT score. Data synthesis was undertaken on the needs, challenges, and characteristics associated with the successful implementation of perioperative registries. Utilising the Directed Content Analysis Method [14], we synthesised and reduced these data into fewer categories based on agreed-upon and predefined criteria.

The agreed-upon and predefined criteria were not part of the search:

### Indications/needs

The primary indication or need to establish a registry in the hospital or region as described by the investigators.

### Successful implementation

The positive outcome or opportunity created by implementing the perioperative registry, as determined by the investigators.

### Challenges in establishing a registry

The reported difficulties in implementing the data registry.

## Results

**Fig. 1:**
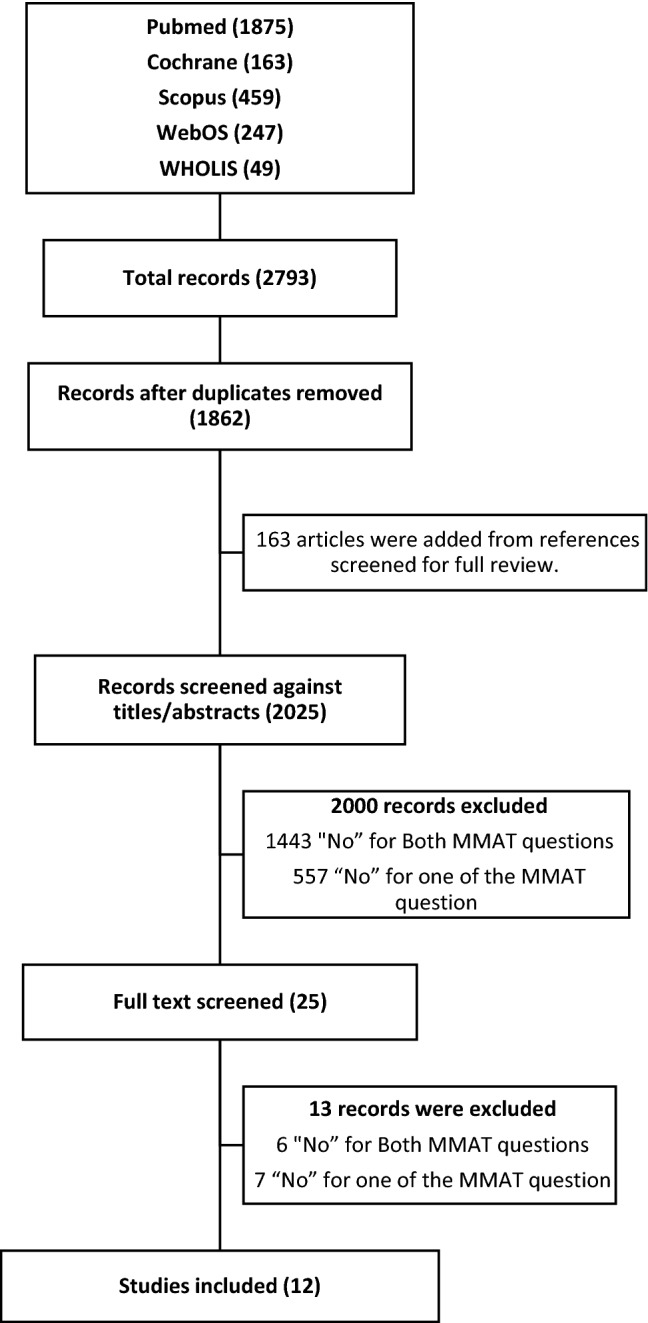
PRISMA flow diagram z

A total of 2793 literature were screened, 931 duplicate records were removed, 163 articles were included from references screened for full review, and 2000 records were excluded based on responses to the screening questions of the MMAT (Fig. 1). Thirteen studies were excluded after a full review as six were negative for both screening questions, and seven were negative for the first MMAT question. The twelve articles with two positive answers to the MMAT screening questions were reviewed.

Of the 12 included studies, four were conducted in South America; five in Africa; two in the Middle East; and one in Asia. They were all carried out within individual countries, with eleven being multi-centre studies. The data software included Research Electronic Data Capture (REDCap) used by three hospitals [15–17]; four further studies used unspecified apps, two of which were hosted in the United States of America (USA), where the principal investigators were located [18–21], one used FILE MAKER Pro [22], and the remaining two registries used locally designed software [23, 24]. The databases were located outside the nations where the data collection was conducted in four sites, mainly in the USA and the remaining sites used local storage [18, 19]. Researchers with specific registry objectives typically initiated implementation efforts (*n* = 10) [15–19, 22–26]. Details of the included studies are shown in Table 1.

**Table 1:** Details of included studies

Title	Publication years	Corresponding author location/country	Source of funding	Type of facility/ies	Volume of data	Surgical specialty	Registry developers
Development, implementation, and evaluation of a hybrid electronic medical record system specifically designed for a developing world surgical service	2014	South Africa (Africa)	Unknown	Gov’t (Single centre)	1114	Surgical (general)	Investigators with specific registry objective
Iranian Joint Registry (Iranian National Hip and Knee Arthroplasty Registry)	2016	Iran (Middle East)	Local (Iran MoH)	Gov’t (national)	Not specified	Orthopaedics (Joint replacement)	Investigators with specific registry objective
Implementing Electronic Surgical Registries in Lower-Middle Income Countries: Experiences in Latin America	2016	USA (North America)	USA NIH	Gov’t (multicentre)	10,000	Surgery -trauma	Investigators with specific registry objective
Perioperative adverse events: critical reading of the data registry used in the surgery department of military hospital Moulay Ismail, Meknes	2016	Morocco (Africa)	Unknown	Gov’t (single centre)	1761	Perioperative adverse event	Trainees (students), supervisors and international collaborators
Challenges and opportunities for effective data collection in global neurosurgery: traumatic brain injury surveillance experience in Malawi	2018	USA (all authors) -Africa	USA	Gov’t (single centre)	33,000	Neurosurgery	Investigators with specific registry objective
Mexican registry of pediatric cardiac surgery. First report	2014	Mexico (South America)	No funding	National	880	Congenital Heart Surgery	Association of Congenital Heart Disease Specialists
National Joint Registry of Iran	2019	Iran (Middle East)	Local (Iran MoH)	National gov’t	Not specified	Orthopaedics (Joint replacement)	Investigators and orthopaedic association
Pakistan Registry of Intensive Care (PRICE): Expanding a lower middle-income, clinician-designed critical care registry in South Asia	2019	UK (Asia)	UK and Sri Lanka	Both gov’t and private	11,000	Critical care	Local critical care society and international collaborators
Collaborative Brazilian pediatric renal transplant registry (CoBrazPed-RTx): A report from 2004 to 2018	2019	Brazil (South America)	No funding	National (both gov’t and private)	2744	Paediatric renal transplant	Investigators with specific registry objective
REPLICCAR II Study: Data quality audit in the Paulista Cardiovascular Surgery Registry	2020	Brazil (South America)	Local (Brazil)	Both gov’t and private	2229	Cardiovascular (CVS)	Investigators with specific registry objective
Postoperative Rheumatic Heart Disease Follow-Up: Creating a National Registry and First Results from Rwanda	2020	Rwanda (Africa)	Local and other partners	National	253	Postop RHD	Investigators with specific registry objective
Addressing priorities for surgical research in Africa: implementation of a multicentre cloud-based peri-operative registry in Ethiopia	2021	Ethiopia (Africa)	UK	Gov’t	> 1748	Perioperative	Investigators with specific registry objective

*CVS* Cardiovascular System, *Gov’t* Governmental, *MOG* Ministry of Health, *NIH* National Institutes of Health, *RHD* Rheumatic Heart Disease, *UK*: United Kingdom, *UDA* United States of America

### Indications for establishing the registry

The indications for setting up a perioperative registry in LIC/LMICs included limited data for determining and evaluating patient outcomes (*n = *7) [16–18, 20, 23–25], understanding the volume of surgeries (*n = *2) [16, 23], recognising the burden of diseases (*n = *3) [18, 22], evaluating economic impact (*n = *2) [24, 25], conducting quality improvement initiatives and research (*n = *6) [17, 20, 21, 23–25], auditing and validation of national statistics [26], and informing national/ global surgical indicators and practice (*n = *3) [17, 20, 24] (Table 2). The lack of context-specific data for linking institutions and developing national, regional, and global networks for research and shared learning, as well as identifying short- and long-term training needs for medical and allied professionals, were described (*n = *2) [17, 20].

**Table 2:** Indications for implementing perioperative registries

Indications (References)	Total (N)
To evaluate the demographics of patients [16, 23]	2
To identify risk factors [17, 23]	3
To predict mortality and morbidity [15]	1
To track surgical volume [17, 24]	2
To evaluate patient outcomes [16–18, 20, 23–25]	7
To understand the burden of surgical diseases [18, 22]	2
To evaluate economic impact [24, 25]	2
To conduct quality improvement initiatives and research [17, 20, 21, 23–25]	7
To develop national, regional, and global collaboration networks [16, 18, 19, 26]	4
To inform policy and practice [16, 19, 24]	3
To digitize paper-based clinical data collection mechanism [18]	1
To introduce a Clinical Decision Support System (CDSS) for junior doctors [21]	1
To establish context-specific data for linking institutions [18, 20]	2
To identify short- and long-term training needs for medical and allied professionals [16]	1
To audit and validate national statistics [26]	1

### Characteristics associated with successful implementation

Characteristics of successful implementation are dependent on the personnel, data handling, and data storage and security (Table 3). Personnel requires the building of local research capacity and establishing a local technical team. Engaging different stakeholders (including the Ministry of Health), creating a sense of ownership and responsibility, following local policies, and obtaining appropriate ethical approval from the hosting country's responsible institution, and appropriate recruitment of data collectors in terms of skill-mix and academic background/ability are all associated with the successful implementation of perioperative registries (*n = *5) [16–19, 24–26]. Additionally, the use of international nomenclature for collecting surgical conditions is also recommended to help and conduct multicentre studies across hospitals that use the same codes [26].

**Table 3:** Characteristics associated with successful implementation

Characteristics associated with successful implementation	Total
*Personnel*	
Engaging different stakeholders [16, 18, 19, 24–26]	6
Providing adequate training for data collectors before commencing data collection [16, 18, 19, 23]	4
Appropriate recruitment of data collectors in terms of skill-mix and academic background/ability [16, 18, 19]	3
*Data handling*	
Obtaining appropriate ethical approval [16–19, 23]	5
Building local research capacity [17, 19, 20, 22]	4
Regular data validation [16, 19, 23]	3
Use of international nomenclature for collecting surgical conditions [26]	1
Establishing a local technical team [17, 18]	2
Direct and indirect auditing of the collected data [17]	1
*Software (mobile applications)*	
Easily adaptable or/and locally made [17, 19, 22, 24]	4
Offline compatibility [17, 19, 20]	3
Limited bandwidth [17]	1
Data storage and security	
Enabling fair principles of data management [17, 20, 22, 23]	4
Secure platform [17, 20]	2
Allowing decentralization [16, 17]	2
Data storage transparency (location of the server, number of people who can access the data) [19]	1
Scalable [17]	1
Cost-effective [17, 19, 22]	1
Automated analysis and public view of core surgical indicators [17]	1

Successful software (applications) associated with high-quality data collection was simple to use, had offline compatibility (*n = *3) [17, 19, 20], limited bandwidth (needs nominal data or Wi-Fi connection) (*n = *1) [18], were easily adaptable, and locally made. Data storage transparency (location of the server, number of people who could access the data), providing a scalable, cost-effective, and secure platform, allowing decentralisation, and enabling FAIR (findability, accessibility, interoperability, and reusability) principles of data sharing were associated with successful data storage (*n = *5) [17, 19, 22, 25].

Automated analysis and public view of core surgical indicators were also associated with successful implementation by aiding quality monitoring and creating a convenient way for academics and policymakers to find aggregate information (*n = *1) [17].

## Challenges

The challenges in the implementation of a clinical registry are shown in Table 4. Data handling challenges included concerns related to trust and security of the data collection software and storage (*n = *3) [15, 17, 20], lack of policy for data management and sharing [16, 19, 22], challenges in data quality including data incompleteness [16, 18, 22, 24], data inaccuracies due to data collectors educational levels [15, 16], fear of prosecution, and the emotional impact of registering adverse events among care providers [21], a lack of willingness by hospitals to share data [26], and poor adherence to data collection in areas where surgeons or perioperative care providers themselves collect or input data [15, 22].

**Table 4:** Challenges of implementing perioperative registries in LMICs

Challenges of implementing perioperative registries in LMICs		Total
*Data handling*		
1.	Trust regarding data collection software and storage [15, 17, 20]	3
2.	Lack of policy for data management and sharing [16, 19, 22]	3
3.	Lack of willingness by hospitals to share data [26]	1
4.	Fear of prosecution, and emotional context for registering adverse events among care providers [21]	1
*Data quality*		
1.	Data incompleteness [16, 18, 22, 24]	4
2.	Data inaccuracy due to data collectors' educational levels [15, 16]	2
3.	Limitation on 30 days patients’ follow-up due to difficulty to re-call patients and confirm data [16, 17]	2
4.	Poor adherence to data collection in areas where surgeons or perioperative care providers themselves collect or input data [22, 25]	2
5.	Underreporting of adverse events and poor adherence to data collection in areas where surgeons or perioperative care providers themselves collect or input data [15, 22]	2
6.	Underreporting of adverse events by surgeons [15]	1
7.	Heterogeneity of results between sites when multiple centres were involved [19]	1
*Funding/finance*		
8.	Insufficient funds to organize a team, recruit and pay data collectors [15, 19, 22]	3
9.	High costs for software support from outside the country due to the lack of an established technical team within the hosting country [16, 20, 22]	3
10.	Rapid depreciation of local currencies resulting in difficulties paying data collector salaries in lower-income countries by investigators from higher-income countries [18]	1
*Technical issues*		
11.	Connectivity issues [19]	1
12.	Power outages [22]	1

Finance-related challenges included insufficient funds to organise a team, build infrastructure, and pay data collectors’ salaries, as well as rapid depreciation of local currencies resulting in difficulties paying data collector salaries in lower-income countries by investigators from higher-income countries [15, 17–19, 22, 23], and high costs for software support from outside the country due to lack of an established technical team within the hosting country (*n = *3) [15, 22, 23]. A lack of adequate funding also contributed to restricted follow-up on data collection resulting in incomplete data and an inefficient work process (*n = *1) [15, 19].

Technical challenges included difficulty with connectivity (*n = *1) [19], power outages (*n = *1) [22], as well as the heterogeneity of results between sites when multiple centres were involved (*n = *1) [19].

## Discussion

In this review, we examined twelve studies conducted in low- and low-middle-income countries in the past 50 years from 1969 to 2021 to better understand the indications, challenges, and characteristics of successful perioperative registries in low-resource settings.

The indications for establishing perioperative registries in LMICs include informing the volume and outcomes of surgeries (*n = *7) [16–18, 20, 23, 24], especially as most of the available data come from developed countries with little engagement with the LMIC data owners [10, 27]. The cost implications of surgery and the extent of catastrophic expenditures have been less understood [17, 24, 25] which has been shown in previous reviews [28], and undertaking collaborative research and quality improvement initiatives have been difficult [17, 20, 21, 24], as it requires extensive time and effort, resulting in higher costs. Furthermore, as outlined in other studies, there are relatively few providers in low-resource settings and there is no established registry network to report the Lancet Commission for Global Surgery indicators consistently even though refinements are suggested [29, 30]. The reasons discussed in this systematic review and supported by other literature suggest that it is possible to establish perioperative registries in low-resource settings.

However, the implementation process is complex and challenging. The main challenge for any registry is to collect useable data, which demands agreement on what to collect and how to collect it and keeping the dataset as small as possible [31]. Unless these principles are achieved, it will be impossible to maintain data quality. As described in this review, most of the registries arose from investigators with specific outcome interests (*n = *10) [15–19, 22–26], which may be helpful in determining and minimising the dataset. The question then arises: what is the most efficient method of data collection?

In low-resource contexts, local health data management policies are sparse; therefore, investigators will be compelled to use various techniques to implement a clinical registry, often resulting in a protracted and laborious procedure [32, 33]. Finding local collaborators, obtaining ethical approval (*n = *5) [15–18, 22], and making it a national project [24, 25] with the involvement of responsible governmental offices such as the Ministry of Health, Ministry of Education, and the National Social Security Administration were all part of the implementation process in most of the successful studies reviewed. In the absence of a data management policy which would expedite the process, acquiring applicable ethical approvals and finding and engaging local stakeholders are all linked to successful perioperative registries in low-resource settings.

When designing software (mobile applications), local constraints such as unreliable connectivity and insufficient power supplies must be considered. As a result, software and programs must be offline compatible, have a low bandwidth requirement, be adaptive, and be user-friendly. Data collection hardware (e.g., tablets and computers) must have a long battery life, particularly when data is collected from remote areas, and data collectors must travel long distances to upload it to the server. REDCap was the most commonly utilised data collection application amongst included studies *n = *3 [15–17], and it can provide the above functionalities. In addition, even though it has not been observed in the studies reviewed, District Health Information 2 (DHIS 2) is free and open-source software that is available and is recommended by the World Health Organisation (WHO) [34].

The next consideration is data storage and security, which should be highly transparent to ensure public trust in the electronic data gathering system. The best data storage solution must be cost-effective, synchronisable, scalable, and accept data in different formats. Finally, it should support the FAIR principles of data sharing [35]. The findings of this study show that cloud storage has significant advantages in terms of the qualities described above, while security issues remain [17]. New research suggests cloud storage combined with Blochian technology may provide more robust security and better deception management in clinical data management [36].

The next stage is to agree on who will collect the data, as the quality of the data was influenced by the training of the data collectors. Data collectors that are academically qualified and well-trained are desirable [13, 20]. With a basic understanding of the data elements provided, medical/health science students, nurses, interns, and registrars (residents) may provide higher quality data [15, 19]. However, surgeons and perioperative care providers appear to be associated with underreporting adverse events in our review (*n = *2) [22, 25], which could be attributed to data collection bias, and this is also reported in high-income countries [37].

Once data collection has commenced, continuous data quality monitoring and regular data quality auditing have proven to be useful [13]. In addition to supporting regular aggregate data monitoring, the inclusion of key local surgical [17], and Lancet Commission for Global Surgery indicators via aggregate dashboard is likely to prove beneficial in making the data monitoring process simple and assisting the public and policymakers in identifying gaps and improving overall care.

Finally, building local teams and giving ownership are also indicated for long-term sustainability [16], particularly when work is led and coordinated outside the hosting country, and this should be considered from the start; this includes building the Information and Communications Technology (ICT) team, data analysis, and the scientific community (perioperative clinicians and researchers), as well as inviting major stakeholders such as the Ministry of Health and professional societies. Everyone should understand that they own the data resource [16, 17], which they can use in accordance with local ethical approval and data management policies. The regular meeting of collaborators to establish standards and share best practices has also proven highly beneficial in high-income countries to improve the quality of care, share responsibility, and obtain sustainable funding sources [5, 38].

There are some limitations to this study. This study excluded grey literature which may have included data relevant to this topic. Additionally, some studies, whilst focused on LMICs, were funded by HICs, hence making the extrapolation of findings focused on LMICs more complex. Furthermore, the search may have missed potential registries by not using the keywords like “anesthesiology” and others, plus the evaluated studies are primarily focused on specific surgical specialties, therefore, the findings from this analysis may not be generalisable to large-scale, all-inclusive perioperative registries, however, as similar challenges and characteristics were identified across studies, it is possible that they are more broadly generalisable.

**Fig. 2:**
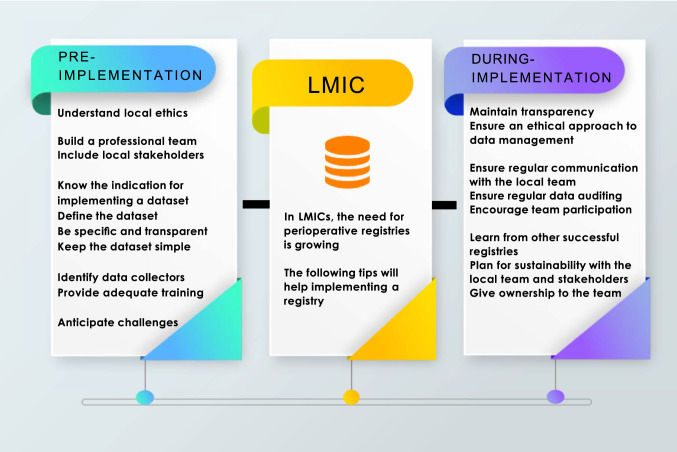
Infographic of characteristics necessary for perioperative registry implementation in LMICs

In conclusion, the goal of a perioperative registry is to generate data to influence and support quality improvement, and national surgical policies; continuously generating clinical data are necessary to represent and lead this effort to support evidence-based collaborations, decisions, and interventions to improve the capacity and quality of care delivered. Artificial intelligence and big data analytics will only be possible if data registries can be routinely established in low-resource environments [39, 40]. This paper provides information on the strategies necessary to achieve this goal. Although some of the challenges mentioned are not unique to low-resource settings, such as finance, data incompleteness, relevance of the variables being collected, most developed nations encounter these challenges when expanding variables or developing more subspecialty surgical registries, in contrast to low-resource settings that have no registries and contribute little to global surgical data [6, 41–43]. LMICs must not fall further behind as new surgical technology innovations are launched. As a result, every effort to establish perioperative registries must continue whilst adhering to local ethical practices and broader practices and should be based on those characteristics associated with successful registry implementation as identified in this review, as summarised in Fig. 2. A meta-analysis and further research are also needed to support this study’s findings and are recommended in the future.

## Supplementary Information

Below is the link to the electronic supplementary material.

Supplementary file1 (PDF 236 kb)
