# Supplementary material for: Indications, Challenges, and Characteristics of Successful Implementation of Perioperative Registries in Low Resource Settings: A Systematic Review
Source: World J Surg. 2023 Jan 19;47(6):1387–96. doi: 10.1007/s00268-023-06909-6 (PMC10156757; doi:10.1007/s00268-023-06909-6)
Supplement: Supplementary file 1 — Supplementary file1 (PDF 236 kb) [file 268_2023_6909_MOESM1_ESM.pdf]

Indications, challenges, and characteristics of successful implementation of perioperative registries in low resource settings: a systematic review

Supplemental material 1: Search strategies

PubMed Database

Search date July 23/2021

Database	MESH	Keywords
**PubMed**	Registries	Registry OR registries
	AND	
	"Perioperative Medicine"[Mesh] OR "Perioperative care"[Mesh] OR "General Surgery"[Mesh]	Perioperative medicine OR peri-operative medicine OR peri-operative care OR Perioperative care OR surgery OR surgical
	AND	
**Filter: LMIC**	TW	Deprived Countries OR Deprived Population OR Deprived Populations OR Developing Countries OR Developing Country OR Developing Economies OR Developing Economy OR Developing Nation OR Developing Nations OR Developing Population OR Developing Populations OR Developing World OR LAMI Countries OR LAMI Country OR Less Developed Countries OR Less Developed Country OR Less Developed Economies OR Less Developed Nation OR Less Developed Nations OR Less Developed World OR Lesser Developed Countries OR Lesser Developed Nations OR LMIC OR LMICS OR Low GDP OR Low GNP OR Low Gross Domestic OR Low Gross National OR Low Income Countries OR Low Income Country OR Low Income Economies OR Low Income Economy OR Low Income Nations OR Low Income Population OR Low Income Populations OR Lower GDP OR lower gross domestic OR Lower Income Countries OR Lower Income Country OR Lower Income Nations OR Lower Income Population OR Lower Income Populations OR Middle Income Countries OR Middle Income Country OR Middle Income Economies OR Middle Income Nation OR Middle Income Nations OR Middle Income Population OR Middle Income Populations OR Poor Countries OR Poor Country OR Poor Economies OR Poor Economy OR Poor Nation OR Poor Nations OR Poor Population OR Poor Populations OR poor world OR Poorer Countries OR Poorer Economies OR Poorer Economy OR Poorer Nations OR Poorer Population OR Poorer Populations OR Third World OR Transitional Countries OR Transitional Country OR Transitional Economies OR Transitional Economy OR Under Developed Countries OR Under Developed Country OR under developed nations OR Under Developed World OR Under Served Population OR Under Served Populations OR Underdeveloped Countries OR Underdeveloped Country OR underdeveloped economies OR underdeveloped nations OR underdeveloped population OR Underdeveloped World OR Underserved Countries OR Underserved Nations OR Underserved Population OR Underserved Populations

	OR	
	TW	Afghanistan OR Albania OR Algeria OR American Samoa OR Angola OR Armenia OR Azerbaijan OR Bangladesh OR Belarus OR Byelarus OR Belorussia OR Belize OR Benin OR Bhutan OR Bolivia OR Bosnia OR Botswana OR Brazil OR Bulgaria OR Burma OR Burkina Faso OR Burundi OR Cabo Verde OR Cape Verde OR Cambodia OR Cameroon OR Central African Republic OR Chad OR China OR Colombia OR Comoros OR Comores OR Comoro OR Congo OR Costa Rica OR Côte d'Ivoire OR Cuba OR Djibouti OR Dominica OR Dominican Republic OR Ecuador OR Egypt OR El Salvador OR Equatorial Guinea OR Eritrea OR Ethiopia OR Fiji OR Gabon OR Gambia OR Gaza OR Georgia OR Georgia Republic OR Ghana OR Grenada OR Grenadines OR Guatemala OR Guinea OR Guinea- Bissau OR Guyana OR Haiti OR Herzegovina OR Hercegovina OR Honduras OR India OR Indonesia OR Iran OR Iraq OR Ivory Coast OR Jamaica OR Jordan OR Kazakhstan OR Kenya OR Kiribati OR Democratic People's Republic of Korea OR Kosovo OR Kyrgyz OR Kirghizia OR Kirghiz OR Kyrgyzstan OR Lao PDR OR Laos OR Lebanon OR Lesotho OR Liberia OR Libya OR Macedonia OR Madagascar OR Malawi OR Malay OR Malaya OR Malaysia OR Maldives OR Mali OR Marshall Islands OR Mauritania OR Mauritius OR Mexico OR Micronesia OR Moldova OR Mongolia OR Montenegro OR Morocco OR Mozambique OR Myanmar OR Namibia OR Nepal OR Nicaragua OR Niger OR Nigeria OR Pakistan OR Palau OR Papua New Guinea OR Paraguay OR Peru OR Philippines OR Principe OR Romania OR Ruanda OR Rwanda OR Samoa OR Sao Tome OR Senegal OR Serbia OR Sierra Leone OR Solomon Islands OR Somalia OR South Africa OR South Sudan OR Sri Lanka OR St Lucia OR St Vincent OR Sudan OR Surinam OR Suriname OR Swaziland OR Syria OR Syrian Arab Republic OR Tajikistan OR Tadzhikistan OR Tajikistan OR Tadzhik OR Tanzania OR Thailand OR Timor OR Togo OR Tonga OR Tunisia OR Turkey OR Turkmen OR Turkmenistan OR Tuvalu OR Uganda OR Ukraine OR Uzbek OR Uzbekistan OR Vanuatu OR Venezuela OR Vietnam OR West Bank OR Yemen OR Zambia OR Zimbabwe
**Final search:** https://www.ncbi.nlm.nih.gov/sites/myncbi/1LwlVrv_cv_5m/collections/60952653/public/		((Deprived Countries[Text Word] OR Deprived Population[Text Word] OR Deprived Populations[Text Word] OR Developing Countries[Text Word] OR Developing Country[Text Word] OR Developing Economies[Text Word] OR Developing Economy[Text Word] OR Developing Nation[Text Word] OR Developing Nations[Text Word] OR Developing Population[Text Word] OR Developing Populations[Text Word] OR Developing World[Text Word] OR LAMI Countries[Text Word] OR LAMI Country[Text Word] OR Less Developed Countries[Text Word] OR Less Developed Country[Text Word] OR Less Developed Economies [Text Word] OR Less Developed Nation[Text Word] OR Less Developed Nations[Text Word] OR Less Developed World[Text Word] OR Lesser Developed Countries[Text Word] OR Lesser

	Developed Nations[Text Word] OR LMIC[Text Word] OR LMICS[Text Word] OR Low GDP[Text Word] OR Low GNP[Text Word] OR Low Gross Domestic[Text Word] OR Low Gross National[Text Word] OR Low Income Countries[Text Word] OR Low Income Country[Text Word] OR Low Income Economies [Text Word] OR Low Income Economy[Text Word] OR Low Income Nations[Text Word] OR Low Income Population[Text Word] OR Low Income Populations[Text Word] OR Lower GDP[Text Word] OR lower gross domestic[Text Word] OR Lower Income Countries[Text Word] OR Lower Income Country[Text Word] OR Lower Income Nations[Text Word] OR Lower Income Population[Text Word] OR Lower Income Populations[Text Word] OR Middle Income Countries[Text Word] OR Middle Income Country[Text Word] OR Middle Income Economies [Text Word] OR Middle Income Nation[Text Word] OR Middle Income Nations[Text Word] OR Middle Income Population[Text Word] OR Middle Income Populations[Text Word] OR Poor Countries[Text Word] OR Poor Country[Text Word] OR Poor Economies [Text Word] OR Poor Economy[Text Word] OR Poor Nation[Text Word] OR Poor Nations[Text Word] OR Poor Population[Text Word] OR Poor Populations[Text Word] OR poor world[Text Word] OR Poorer Countries[Text Word] OR Poorer Economies [Text Word] OR Poorer Economy[Text Word] OR Poorer Nations[Text Word] OR Poorer Population[Text Word] OR Poorer Populations[Text Word] OR Third World[Text Word] OR Transitional Countries[Text Word] OR Transitional Country[Text Word] OR Transitional Economies[Text Word] OR Transitional Economy[Text Word] OR Under Developed Countries[Text Word] OR Under Developed Country[Text Word] OR under developed nations[Text Word] OR Under Developed World[Text Word] OR Under Served Population[Text Word] OR Under Served Populations[Text Word] OR Underdeveloped Countries[Text Word] OR Underdeveloped Country[Text Word] OR underdeveloped economies[Text Word] OR underdeveloped nations[Text Word] OR underdeveloped population[Text Word] OR Underdeveloped World[Text Word] OR Underserved Countries[Text Word] OR Underserved Nations[Text Word] OR Underserved Population[Text Word] OR Underserved Populations[Text Word]) OR (Afghanistan[Text Word] OR Albania[Text Word] OR Algeria[Text Word] OR American Samoa[Text Word] OR Angola[Text Word] OR Armenia[Text Word] OR Azerbaijan[Text Word] OR Bangladesh[Text Word] OR Belarus[Text Word] OR Byelarus[Text Word] OR Belorussia[Text Word] OR Belize[Text Word] OR Benin[Text Word] OR Bhutan[Text Word] OR Bolivia[Text Word] OR Bosnia[Text Word] OR Botswana[Text Word] OR Brazil[Text Word] OR Bulgaria[Text Word] OR Burma[Text Word] OR Burkina Faso[Text Word] OR Burundi[Text Word] OR Cabo Verde[Text Word] OR Cape Verde[Text Word] OR Cambodia[Text Word] OR Cameroon[Text Word] OR Central African Republic[Text Word] OR Chad[Text Word] OR China[Text Word] OR Colombia[Text Word] OR Comoros[Text Word] OR Comores[Text Word] OR Comoro[Text

	Word] OR Congo[Text Word] OR Costa Rica[Text Word] OR Côte d'Ivoire[Text Word] OR Cuba[Text Word] OR Djibouti[Text Word] OR Dominica[Text Word] OR Dominican Republic[Text Word] OR Ecuador[Text Word] OR Egypt[Text Word] OR El Salvador[Text Word] OR Equatorial Guinea[Text Word] OR Eritrea[Text Word] OR Ethiopia[Text Word] OR Fiji[Text Word] OR Gabon[Text Word] OR Gambia[Text Word] OR Gaza[Text Word] OR Georgia[Text Word] OR Georgia Republic[Text Word] OR Ghana[Text Word] OR Grenada[Text Word] OR Grenadines[Text Word] OR Guatemala[Text Word] OR Guinea[Text Word] OR Guinea-Bissau[Text Word] OR Guyana[Text Word] OR Haiti[Text Word] OR Herzegovina[Text Word] OR Hercegovina[Text Word] OR Honduras[Text Word] OR India[Text Word] OR Indonesia[Text Word] OR Iran[Text Word] OR Iraq[Text Word] OR Ivory Coast[Text Word] OR Jamaica[Text Word] OR Jordan[Text Word] OR Kazakhstan[Text Word] OR Kenya[Text Word] OR Kiribati[Text Word] OR Democratic People's Republic of Korea[Text Word] OR Kosovo[Text Word] OR Kyrgyz[Text Word] OR Kirghizia[Text Word] OR Kirghiz[Text Word] OR Kyrgyzstan[Text Word] OR Lao PDR[Text Word] OR Laos[Text Word] OR Lebanon[Text Word] OR Lesotho[Text Word] OR Liberia[Text Word] OR Libya[Text Word] OR Macedonia[Text Word] OR Madagascar[Text Word] OR Malawi[Text Word] OR Malay[Text Word] OR Malaya[Text Word] OR Malaysia[Text Word] OR Maldives[Text Word] OR Mali[Text Word] OR Marshall Islands[Text Word] OR Mauritania[Text Word] OR Mauritius[Text Word] OR Mexico[Text Word] OR Micronesia[Text Word] OR Moldova[Text Word] OR Mongolia[Text Word] OR Montenegro[Text Word] OR Morocco[Text Word] OR Mozambique[Text Word] OR Myanmar[Text Word] OR Namibia[Text Word] OR Nepal[Text Word] OR Nicaragua[Text Word] OR Niger[Text Word] OR Nigeria [Text Word] OR Pakistan [Text Word] OR Palau[Text Word] OR Papua New Guinea[Text Word] OR Paraguay[Text Word] OR Peru [Text Word] OR Philippines[Text Word] OR Principe[Text Word] OR Romania[Text Word] OR Ruanda[Text Word] OR Rwanda[Text Word] OR Samoa[Text Word] OR Sao Tome[Text Word] OR Senegal[Text Word] OR Serbia[Text Word] OR Sierra Leone[Text Word] OR Solomon Islands[Text Word] OR Somalia[Text Word] OR South Africa[Text Word] OR South Sudan[Text Word] OR Sri Lanka[Text Word] OR St Lucia[Text Word] OR St Vincent OR[Text Word])) AND (((("Perioperative Care"[Mesh]) OR (peri-operative care OR Perioperative care)) OR (("Perioperative Medicine"[Mesh]) OR (Perioperative medicine OR peri-operative medicine))) AND (("Registries"[Mesh]) OR (registry OR registries))) Sort by: Most Recent

Cochrane Database Search

Search conducted 9 August 2021

https://www.cochranelibrary.com/advanced-search/search-manager?search=5700006

ID	Search	Hits
**#1**	(Registry OR registries):ti,ab,kw AND ("Perioperative medicine" OR "peri-operative medicine" OR "peri-operative care" OR "perioperative care" OR surgery OR surgical):ti,ab,kw (Word variations have been searched)	4042
**#2**	(Afghanistan OR Albania OR Algeria OR “American Samoa” OR Angola OR Armenia OR Azerbaijan OR Bangladesh OR Belarus OR Byelarus OR Belorussia OR Belize OR Benin OR Bhutan OR Bolivia OR Bosnia OR Botswana OR Brazil OR Bulgaria OR Burma OR “Burkina Faso” OR Burundi OR “Cabo Verde” OR “Cape Verde” OR Cambodia OR Cameroon OR “Central African Republic” OR Chad OR China OR Colombia OR Comoros OR Comores OR Comoro OR Congo OR “Costa Rica” OR “Côte d'Ivoire” OR Cuba OR “Democratic People’s Republic of Korea” OR Djibouti OR Dominica OR “Dominican Republic” OR Ecuador OR Egypt OR “El Salvador” OR Eritrea OR Ethiopia OR “Equatorial Guinea” OR Fiji OR Gabon OR Gambia OR Gaza OR “Georgia Republic” OR Georgia OR Ghana OR Grenada OR Grenadines OR Guatemala OR Guinea OR “Guinea Bissau” OR Guyana OR Haiti OR Herzegovina OR Hercegovina OR Honduras OR India OR Indonesia OR Iran OR Iraq OR “Ivory Coast” OR Jamaica OR Jordan OR Kazakhstan OR Kenya OR Kiribati OR Korea OR Kosovo OR Kyrgyz OR Kirghizia OR Kirghiz OR Kyrgyzstan OR “Lao PDR” OR Laos OR Lebanon OR Lesotho OR Liberia OR Libya OR Macedonia OR Madagascar OR Malawi OR Malay OR Malaya OR Malaysia OR Maldives OR Mali OR “Marshall Islands” OR Mauritania OR Mauritius OR Mexico OR Micronesia OR Moldova OR Mongolia OR Montenegro OR Morocco OR Mozambique OR Myanmar OR Namibia OR Nepal OR Nicaragua OR Niger OR Nigeria OR Pakistan OR Palau OR “Papua New Guinea” OR Paraguay OR Peru OR Philippines OR Principe OR Romania OR Rwanda OR Ruanda OR Samoa OR “Sao Tome” OR Senegal OR Serbia OR “Sierra Leone” OR “Solomon Islands” OR Somalia OR “South Africa” OR “South Sudan” OR “Sri Lanka” OR “St Lucia” OR “St Vincent” OR Sudan OR Surinam OR Suriname OR Swaziland OR Syria OR “Syrian Arab Republic” OR Tajikistan OR Tadzhikistan OR Tajikistan OR Tadzhik OR Tanzania OR Thailand OR Timor OR Togo OR Tonga OR Tunisia OR Turkey OR Turkmen OR Turkmenistan OR Tuvalu OR Uganda OR Ukraine OR Uzbek OR Uzbekistan OR Vanuatu OR Venezuela OR Vietnam OR “West Bank” OR Yemen OR Zambia OR Zimbabwe):ti,ab,kw (Word variations have been searched)	96568

**#3**	(Deprived Countries OR "Deprived Population" OR "Deprived Populations" OR "Developing Countries" OR "Developing Country" OR "Developing Economies" OR "Developing Economy" OR "Developing Nation" OR "Developing Nations" OR "Developing Population" OR "Developing Populations" OR "Developing World" OR "LAMI Countries" OR "LAMI Country" OR "Less Developed Countries" OR "Less Developed Country" OR "Less Developed Economies" OR "Less Developed Nation" OR "Less Developed Nations" OR "Less Developed World" OR "Lesser Developed Countries" OR "Lesser Developed Nations" OR LMIC OR LMICS OR Low GDP OR "Low GNP" OR "Low Gross Domestic" OR "Low Gross National" OR "Low Income Countries" OR "Low Income Country" OR "Low Income Economies" OR "Low Income Economy" OR "Low Income Nations" OR "Low Income Population" OR "Low Income Populations" OR "Lower GDP" OR "lower gross domestic" OR "Lower Income Countries" OR "Lower Income Country" OR "Lower Income Nations" OR "Lower Income Population" OR "Lower Income Populations" OR "Middle Income Countries" OR "Middle Income Country" OR "Middle Income Economies" OR "Middle Income Nation" OR "Middle Income Nations" OR "Middle Income Population" OR "Middle Income Populations" OR "Poor Countries" OR "Poor Country" OR "Poor Economies" OR "Poor Economy" OR "Poor Nation" OR "Poor Nations" OR "Poor Population" OR "Poor Populations" OR "poor world" OR "Poorer Countries" OR "Poorer Economies" OR "Poorer Economy" OR "Poorer Nations" OR "Poorer Population" OR "Poorer Populations" OR "Third World" OR "Transitional Countries" OR "Transitional Country" OR "Transitional Economies" OR "Transitional Economy" OR "Under Developed Countries" OR "Under Developed Country" OR "under developed nations" OR "Under Developed World" OR "Under Served Population" OR "Under Served Populations" OR "Underdeveloped Countries" OR "Underdeveloped Country" OR "underdeveloped economies" OR "underdeveloped nations" OR "underdeveloped population" OR "Underdeveloped World" OR "Underserved Countries" OR "Underserved Nations" OR "Underserved Population" OR "Underserved Populations"):ti,ab,kw (Word variations have been searched)	9065
**#4**	#2 OR #3	100762
**#5**	#1 AND #4	540
**Limited to Cochrane Reviews**		163

Scopus includes EMBASE
Search conducted 7 August 2021

Search	Query	Records retrieved
#1	TITLE-ABS-KEY (registry OR registries)	211 827
#2	TITLE-ABS-KEY (“Perioperative medicine” OR “peri-operative medicine” OR “peri-operative care” OR “Perioperative care” OR surgery OR surgical)	3 241 897
#3	#1 AND #2	38,060
#4	TITLE-ABS-KEY (“Deprived Country“ OR “Deprived Countries” OR “Deprived Population” OR “Deprived Populations” OR “Developing Countries” OR “Developing Country” OR “Developing Economies” OR “Developing Economy” OR “Developing Nation” OR “Developing Nations” OR “Developing Population” OR “Developing Populations” OR “Developing World” OR “LAMI Countries” OR “LAMI Country” OR “Less Developed Countries” OR “Less Developed Country” OR “Less Developed Economies” OR “Less Developed Nation” OR “Less Developed Nations” OR “Less Developed World” OR “Lesser Developed Countries” OR “Lesser Developed Nations” OR LMIC OR LMICS OR “Low GDP” OR “Low GNP” OR “Low Gross Domestic” OR “Low Gross National” OR “Low Income Countries” OR “Low Income Country” OR “Low Income Economies” OR “Low Income Economy” OR “Low Income Nations” OR “Low Income Population” OR “Low Income Populations” OR “Lower GDP” OR “Lower Gross Domestic” OR “Lower Income Countries” OR “Lower Income Country” OR “Lower Income Nations” OR “Lower Income Population” OR “Lower Income Populations” OR “Middle Income Countries” OR “Middle Income Country” OR “Middle Income Economies” OR “Middle Income Nation” OR “Middle Income Nations” OR “Middle Income Population” OR “Middle Income Populations” OR “Poor Countries” OR “Poor Country” OR “Poor Economies” OR “Poor Economy” OR “Poor Nation” OR “Poor Nations” OR “Poor Population” OR “Poor Populations” OR “Poor World” OR “Poorer Countries” OR “Poorer Economies” OR “Poorer Economy” OR “Poorer Nations” OR “Poorer Population” OR “Poorer Populations” OR “Third World” OR “Transitional Countries” OR “Transitional Country” OR “Transitional Economies” OR “Transitional Economy” OR “Under Developed Countries” OR “Under Developed Country” OR “Under Developed Nations” OR “Under Developed World” OR “Under Served Population” OR “Under Served Populations” OR “Underdeveloped Countries” OR “Underdeveloped Country” OR “Underdeveloped Economies” OR “Underdeveloped Nations” OR “Underdeveloped Population” OR “Underdeveloped World” OR “Underserved Countries” OR “Underserved Nations” OR “Underserved Population” OR “Underserved Populations”)	383 798
#5	TITLE-ABS-KEY (Afghanistan OR Albania OR Algeria OR “American Samoa” OR Angola OR Armenia OR Azerbaijan OR Bangladesh OR Belarus OR Byelarus OR Belorussia OR Belize OR Benin OR Bhutan OR Bolivia OR Bosnia OR Botswana OR Brazil OR Bulgaria OR Burma OR “Burkina Faso” OR Burundi OR “Cabo Verde” OR “Cape Verde” OR	4 612 856

	Cambodia OR Cameroon OR “Central African Republic” OR Chad OR China OR Colombia OR Comoros OR Comores OR Comoro OR Congo OR “Costa Rica” OR “Côte d'Ivoire” OR Cuba OR “Democratic People’s Republic of Korea” OR Djibouti OR Dominica OR “Dominican Republic” OR Ecuador OR Egypt OR “El Salvador” OR Eritrea OR Ethiopia OR “Equatorial Guinea” OR Fiji OR Gabon OR Gambia OR Gaza OR “Georgia Republic” OR Georgia OR Ghana OR Grenada OR Grenadines OR Guatemala OR Guinea OR “Guinea Bissau” OR Guyana OR Haiti OR Herzegovina OR Hercegovina OR Honduras OR India OR Indonesia OR Iran OR Iraq OR “Ivory Coast” OR Jamaica OR Jordan OR Kazakhstan OR Kenya OR Kiribati OR Korea OR Kosovo OR Kyrgyz OR Kirghizia OR Kirghiz OR Kyrgyzstan OR “Lao PDR” OR Laos OR Lebanon OR Lesotho OR Liberia OR Libya OR Macedonia OR Madagascar OR Malawi OR Malay OR Malaya OR Malaysia OR Maldives OR Mali OR “Marshall Islands” OR Mauritania OR Mauritius OR Mexico OR Micronesia OR Moldova OR Mongolia OR Montenegro OR Morocco OR Mozambique OR Myanmar OR Namibia OR Nepal OR Nicaragua OR Niger OR Nigeria OR Pakistan OR Palau OR “Papua New Guinea” OR Paraguay OR Peru OR Philippines OR Principe OR Romania OR Rwanda OR Ruanda OR Samoa OR “Sao Tome” OR Senegal OR Serbia OR “Sierra Leone” OR “Solomon Islands” OR Somalia OR “South Africa” OR “South Sudan” OR “Sri Lanka” OR “St Lucia” OR “St Vincent” OR Sudan OR Surinam OR Suriname OR Swaziland OR Syria OR “Syrian Arab Republic” OR Tajikistan OR Tadzhikistan OR Tajikistan OR Tadzhik OR Tanzania OR Thailand OR Timor OR Togo OR Tonga OR Tunisia OR Turkey OR Turkmen OR Turkmenistan OR Tuvalu OR Uganda OR Ukraine OR Uzbek OR Uzbekistan OR Vanuatu OR Venezuela OR Vietnam OR “West Bank” OR Yemen OR Zambia OR Zimbabwe)	
#6	#4 OR #5	4 809 910
#7	#3 AND #6	2 305
#8	INDEX(Medline)	28 010 453
#9	#7 AND NOT #8	459

SciELO Citation Index via Web of Science
Search conducted 7 August 2021

https://www.webofscience.com/wos/scielo/summary/f388e026-a9bc-4072-a1a8-23200a9a18f9-0374055f/relevance/1

Search	Query	Records retrieved
#1	Registry OR registries (Topic)	2 419
#2	“Perioperative medicine” OR “peri-operative medicine” OR “peri-operative care” OR “Perioperative care” OR surgery OR surgical (Topic)	38 064
#3	#1 AND #2	247

Web of Science Core Collection
Search conducted 7 August 2021

https://www.webofscience.com/wos/woscc/summary/6c4181b0-b8b0-4847-9052-541f0cc27eaa-0373bb18/relevance/1

Search	Query	Records retrieved
#1	Registry OR registries (Topic)	169 593
#2	“Perioperative medicine” OR “peri-operative medicine” OR “peri-operative care” OR “Perioperative care” OR surgery OR surgical (Topic)	1 720 896
#3	#1 AND #2	26 299
#4	(TS=) “Deprived Country“ OR “Deprived Countries” OR “Deprived Population” OR “Deprived Populations” OR “Developing Countries” OR “Developing Country” OR “Developing Economies” OR “Developing Economy” OR “Developing Nation” OR “Developing Nations” OR “Developing Population” OR “Developing Populations” OR “Developing World” OR “LAMI Countries” OR “LAMI Country” OR “Less Developed Countries” OR “Less Developed Country” OR “Less Developed Economies” OR “Less Developed Nation” OR “Less Developed Nations” OR “Less Developed World” OR “Lesser Developed Countries” OR “Lesser Developed Nations” OR LMIC OR LMICS OR “Low GDP” OR “Low GNP” OR “Low Gross Domestic” OR “Low Gross National” OR “Low Income Countries” OR “Low Income Country” OR “Low Income Economies” OR “Low Income Economy” OR “Low Income Nations” OR “Low Income Population” OR “Low Income Populations” OR “Lower GDP” OR “Lower Gross Domestic” OR “Lower Income Countries” OR “Lower Income Country” OR “Lower Income Nations” OR “Lower Income Population” OR “Lower Income Populations” OR “Middle Income Countries” OR “Middle Income Country” OR “Middle Income Economies” OR “Middle Income Nation” OR “Middle Income Nations” OR “Middle Income Population” OR “Middle Income Populations” OR “Poor Countries” OR “Poor Country” OR “Poor Economies” OR “Poor Economy” OR “Poor Nation” OR “Poor Nations” OR “Poor Population” OR “Poor Populations” OR “Poor World” OR “Poorer Countries” OR “Poorer Economies” OR “Poorer Economy” OR “Poorer Nations” OR “Poorer Population” OR “Poorer Populations” OR “Third World” OR “Transitional Countries” OR “Transitional Country” OR “Transitional Economies” OR “Transitional Economy” OR “Under Developed Countries” OR “Under Developed Country” OR “Under Developed Nations” OR “Under Developed World” OR “Under Served Population” OR “Under Served Populations” OR “Underdeveloped Countries” OR “Underdeveloped Country” OR “Underdeveloped Economies” OR “Underdeveloped Nations” OR “Underdeveloped Population” OR “Underdeveloped World” OR “Underserved Countries” OR “Underserved Nations” OR “Underserved Population” OR “Underserved Populations”	204 944
#5	(TS=) Afghanistan OR Albania OR Algeria OR “American Samoa” OR Angola OR Armenia OR Azerbaijan OR Bangladesh OR Belarus OR Byelarus OR Belorussia OR Belize OR Benin OR Bhutan OR Bolivia OR Bosnia OR Botswana OR Brazil OR Bulgaria OR Burma OR “Burkina Faso” OR Burundi OR “Cabo Verde” OR “Cape Verde” OR Cambodia OR Cameroon OR	2 960 856

	“Central African Republic” OR Chad OR China OR Colombia OR Comoros OR Comores OR Comoro OR Congo OR “Costa Rica” OR “Côte d'Ivoire” OR Cuba OR “Democratic People’s Republic of Korea” OR Djibouti OR Dominica OR “Dominican Republic” OR Ecuador OR Egypt OR “El Salvador” OR Eritrea OR Ethiopia OR “Equatorial Guinea” OR Fiji OR Gabon OR Gambia OR Gaza OR “Georgia Republic” OR Georgia OR Ghana OR Grenada OR Grenadines OR Guatemala OR Guinea OR “Guinea Bissau” OR Guyana OR Haiti OR Herzegovina OR Hercegovina OR Honduras OR India OR Indonesia OR Iran OR Iraq OR “Ivory Coast” OR Jamaica OR Jordan OR Kazakhstan OR Kenya OR Kiribati OR Korea OR Kosovo OR Kyrgyz OR Kirghizia OR Kirghiz OR Kyrgyzstan OR “Lao PDR” OR Laos OR Lebanon OR Lesotho OR Liberia OR Libya OR Macedonia OR Madagascar OR Malawi OR Malay OR Malaya OR Malaysia OR Maldives OR Mali OR “Marshall Islands” OR Mauritania OR Mauritius OR Mexico OR Micronesia OR Moldova OR Mongolia OR Montenegro OR Morocco OR Mozambique OR Myanmar OR Namibia OR Nepal OR Nicaragua OR Niger OR Nigeria OR Pakistan OR Palau OR “Papua New Guinea” OR Paraguay OR Peru OR Philippines OR Principe OR Romania OR Rwanda OR Ruanda OR Samoa OR “Sao Tome” OR Senegal OR Serbia OR “Sierra Leone” OR “Solomon Islands” OR Somalia OR “South Africa” OR “South Sudan” OR “Sri Lanka” OR “St Lucia” OR “St Vincent” OR Sudan OR Surinam OR Suriname OR Swaziland OR Syria OR “Syrian Arab Republic” OR Tajikistan OR Tadzhikistan OR Tajikistan OR Tadzhik OR Tanzania OR Thailand OR Timor OR Togo OR Tonga OR Tunisia OR Turkey OR Turkmen OR Turkmenistan OR Tuvalu OR Uganda OR Ukraine OR Uzbek OR Uzbekistan OR Vanuatu OR Venezuela OR Vietnam OR “West Bank” OR Yemen OR Zambia OR Zimbabwe	
#6	#4 OR #5	3 074 443
#7	#3 AND #6	1 268
